# Richter’s Hernia With Bowel Perforation in the Umbilical Region: A Unique Pediatric Case

**DOI:** 10.7759/cureus.80062

**Published:** 2025-03-04

**Authors:** Alsadig Suliman, Hussein Elfaki, Ali Mohammed Ibrahim

**Affiliations:** 1 General Surgery, Sudan Medical Specialization Board, Wad Madani, SDN; 2 General Surgery, University of Gezira, Wad Madani, SDN

**Keywords:** antimesenteric side, congenital umbilical hernia, pediatric hernia, richter's hernia, silent perforation of bowel

## Abstract

Richter’s hernia is a rare condition in which only a portion of the bowel’s antimesenteric border becomes incarcerated, often leading to ischemia and perforation without complete obstruction. While commonly seen in elderly patients, pediatric cases, especially congenital umbilical presentations, are rare. We present a case of a four-year-old female with a three-day history of crampy peri-umbilical pain, a non-reducible right paraumbilical bulge, and intermittent vomiting. Despite the patient continuing to pass stool, intraoperative findings revealed an entrapped antimesenteric side of the distal ileal loop, confirming Richter’s hernia with a perforation inside the hernia sac. The perforation was debrided and closed with primary suturing. The hernia was repaired using a double-layer anatomical closure, and the postoperative course was uneventful. This case highlights the deceptive nature of Richter’s hernia and the importance of early recognition, particularly in pediatric congenital umbilical hernias, to prevent severe complications.

## Introduction

Richter’s hernia is characterized by the partial protrusion or strangulation of the antimesenteric border of the bowel through a defect in the abdominal wall [1]. While it primarily affects elderly patients between 60 and 80 years of age, it can occur at any stage of life. In recent years, its incidence has increased, particularly following minimally invasive hernia repairs, where small defect entrapment and trocar site hernias have been implicated [2].

The earliest known case of Richter’s hernia was described by Fabricius Hildanus in 1606, with August Gottlieb Richter formally defining the condition in 1785 [1]. Due to its uncommon occurrence, the available medical literature mainly consists of individual case reports and small case series. The total number of documented cases remains unclear, as new cases continue to be reported. A 2000 review by Steinke and Zellweger analyzed 18 cases treated in a single hospital, emphasizing the distinctive nature of this condition even in specialized centers [3].

Clinically, nausea and vomiting may be present, though these symptoms are generally less common and less severe than in traditional bowel strangulation [4]. If left untreated, Richter’s hernia can lead to serious complications, including bowel gangrene, perforation, post-necrotic abscess formation, enterocutaneous fistula, and potentially fatal outcomes [2]. Richter’s umbilical hernia in pediatric patients represents a unique clinical entity, making each case a noteworthy educational opportunity [1-3]. This report presents an exceptional case of a four-year-old Sudanese female with an incarcerated congenital Richter’s umbilical hernia, present since birth.

## Case presentation

A four-year-old female presented with a three-day history of intermittent abdominal discomfort, as evidenced by irritability and episodes of crying, along with a non-reducible right paraumbilical bulge and episodes of vomiting ingested matter (two to three times per day). There was no fever, abdominal distension, or signs of systemic infection, and the child continued to pass stools and flatus without a significant change in bowel habits. A similar episode had occurred four months earlier, for which she was managed conservatively at the emergency department (ED) and discharged with an appointment for elective surgery. However, she was lost to follow-up. The patient had a history of a paraumbilical bulge since birth, which increased with straining and crying but spontaneously reduced at rest and during sleep.

On physical examination, her vital signs were within normal limits, with a pulse rate of 98 beats per minute, respiratory rate of 23 breaths per minute, and temperature of 37.5°C. Abdominal examination revealed normal movement with respiration and normoactive bowel sounds. A 4×4 cm oval, firm, tender, non-reducible mass was palpated over the right paraumbilical region, without a detectable impulse during crying. The overlying skin was hyperpigmented but not warm or erythematous. Laboratory investigations showed a white blood cell (WBC) count of 7,150/mm³ (reference range: 5,000-15,000/mm³ for children), hematocrit (HCT) of 43.1% (reference range: 30-40% for children), and platelet count of 442,000/mm³ (reference range: 150,000-450,000/mm³ for children). An abdominal ultrasound (US) performed eight months earlier had revealed an umbilical defect measuring 1.8 cm with protruding mesenteric fat. However, an abdominal computed tomography (CT) was not performed due to unavailability. Given the clinical presentation and to prevent further loss to follow-up, an urgent surgical intervention was deemed necessary.

**Figure 1:**
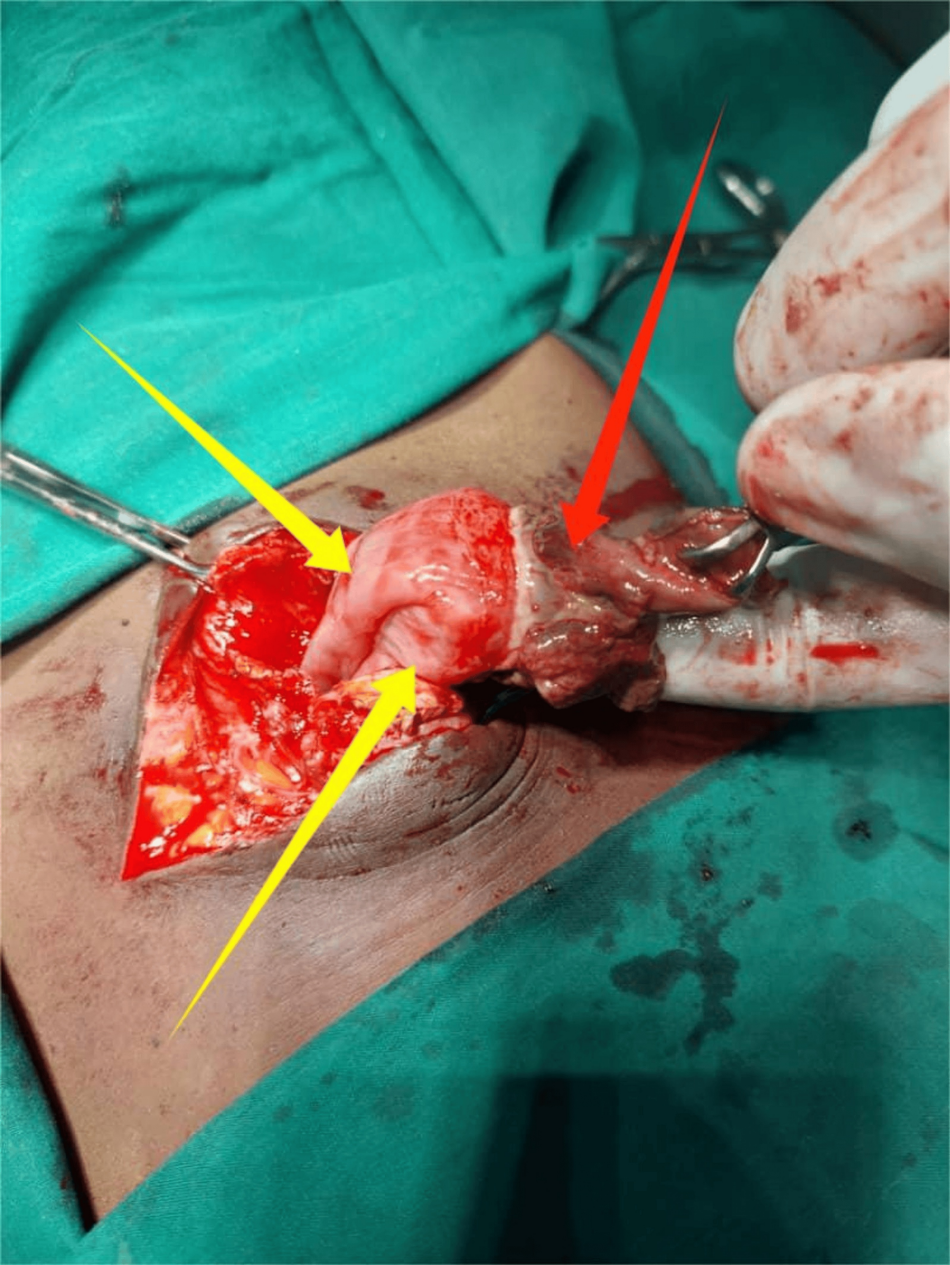
Incarcerated umbilical Richter’s hernia. Yellow arrows highlight the herniated bowel, while the red arrow indicates adhesions between the sac and a knuckle of the distal ileum.

**Figure 2:**
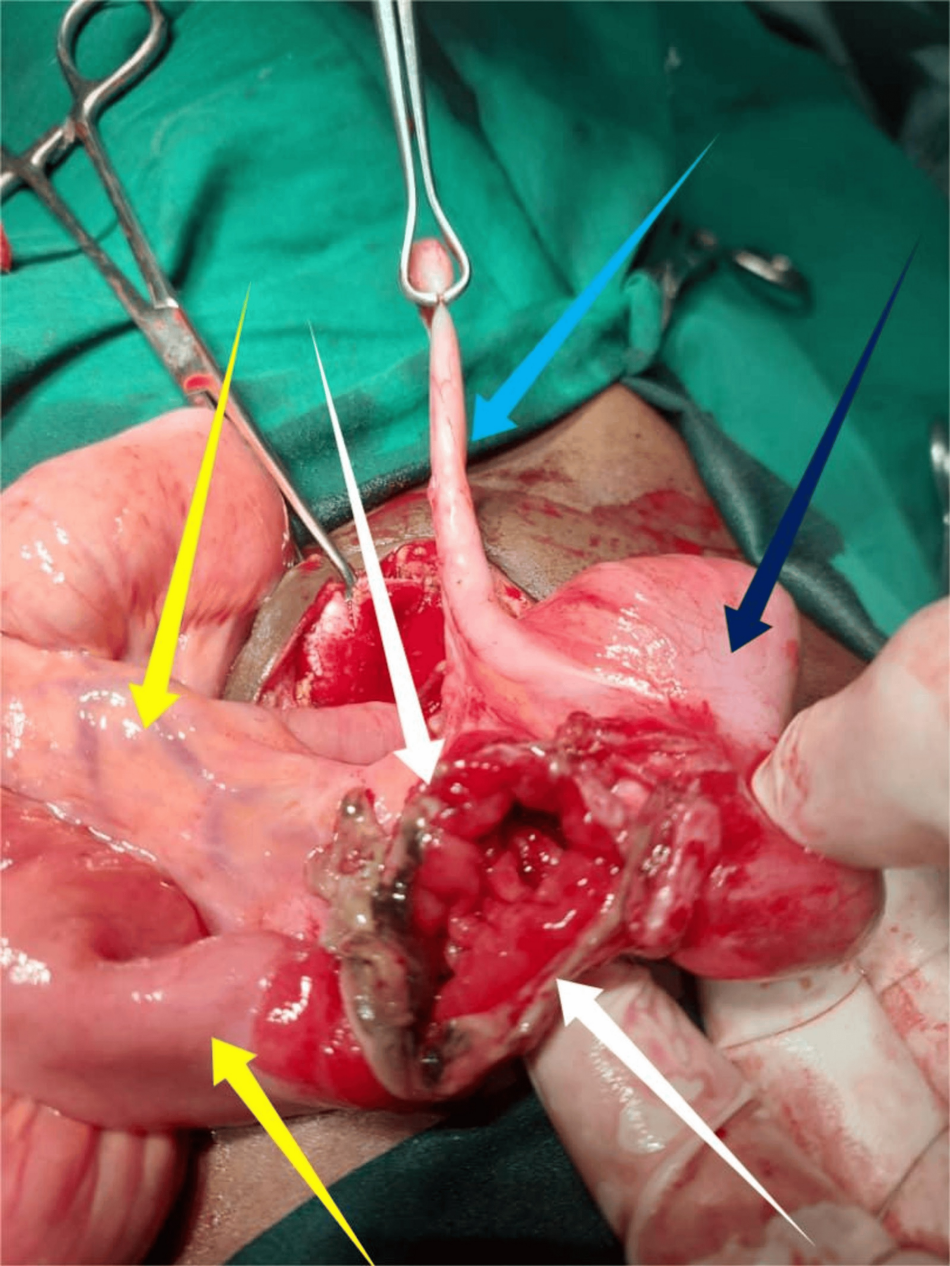
Richter’s hernia with ileal perforation. White arrows indicate the perforated anti-mesenteric ileum, yellow arrows show normal bowel loops, light blue arrow marks the appendix, and dark blue arrow points to the cecum.

Under general anesthesia (GA), with the patient in a supine position, the procedure was performed via a supra-umbilical incision by a senior surgeon. Intraoperative findings confirmed Richter’s hernia, with a loop of the distal ileum entrapped in the umbilical hernia sac on the anti-mesenteric border (Figure 1). The hernia sac contained dark hemorrhagic fluid, with adhesions between the sac and the bowel. A knuckle of the distal ileum was released from its incarceration, revealing a 3 cm perforation within the incarcerated segment (Figure 2). The affected bowel was refashioned, and primary repair was performed. The hernia was repaired using absorbable sutures with a double-layer anatomical repair. The abdominal wall defect measured approximately 2.0 cm, and the procedure was completed without complications.

Postoperatively, the patient remained nil per os (NPO) for 24 hours and received alternating intravenous (IV) fluids. An IV antibiotic was administered, along with appropriate pain management. The postoperative course was uneventful, and the patient was discharged on the third postoperative day. Follow-up visits at the surgical referral clinic over two months showed no postoperative complications.

## Discussion

Richter’s hernia is a rare form of bowel herniation where only a portion of the antimesenteric wall is involved, often leading to ischemia and perforation without causing complete intestinal obstruction [5]. In this case, the patient presented with a three-day history of a non-reducible umbilical bulge and recurrent vomiting. Despite skin discoloration over the hernia, the continued passage of stool and flatus initially reduced clinical suspicion of strangulation and perforation. However, this highlights a key diagnostic pitfall, as partial bowel incarceration in Richter’s hernia does not always cause complete obstruction. This deceptive presentation can delay recognition, leading to severe complications, as observed in our case. Intraoperatively, the diagnosis of Richter’s hernia was confirmed, with an entrapped loop of the distal ileum and a 3 cm perforation at the incarcerated segment. This case underscores the need for heightened clinical suspicion, particularly in pediatric patients, even when classical signs of obstruction are absent.

Richter’s hernia has been reported in various anatomical locations, with the femoral ring being the most frequent site (71%), followed by the deep inguinal ring (23%) and ventral or umbilical hernias (6%) [4]. The distal ileum is the most frequently involved segment of the bowel in cases of entrapment [6]. In contrast to the majority of reported cases, our case involves a congenital umbilical Richter’s hernia in a pediatric patient, which is a particularly uncommon presentation. Additionally, atypical cases have been reported, including Richter’s hernia as a complication of colonoscopy [7]. Other cases have been initially misdiagnosed as groin abscesses, only to be correctly identified after the appearance of feculent discharge [8]. These examples illustrate the diagnostic challenges associated with this condition and emphasize the need for heightened clinical suspicion.

Although Richter’s hernia is most commonly seen in adults, it has also been reported in neonates with small bowel obstruction. A 1986 series at the Royal Aberdeen Children's Hospital described three neonates who presented with bilious vomiting and abdominal distension, all of whom were later diagnosed with Richter’s hernia at the internal inguinal ring during laparotomy [9]. All cases had viable bowel, and suture closure of the hernial sac resulted in successful recovery without recurrence [10]. In contrast, our case involved a congenital umbilical Richter’s hernia that progressed to incarceration and perforation, necessitating urgent surgery. A prior incarceration episode four months earlier was managed conservatively, but loss to follow-up delayed intervention, leading to ischemia and perforation. This highlights the importance of early elective repair in pediatric hernia cases to prevent severe complications.

The surgical approach to Richter’s hernia depends on the size of the defect and the viability of the incarcerated bowel. If bowel necrosis is present, resection with primary anastomosis is required alongside hernia repair. Small defects can often be closed primarily with sutures, whereas larger or more complex defects may necessitate prosthetic mesh reinforcement, particularly in adult patients [11]. According to Skandalakis et al., the gold standard for Richter’s hernia repair is the preperitoneal approach, followed by laparotomy and bowel resection if perforation is suspected [1]. In more challenging cases, additional techniques such as omental reinforcement, peritoneal patches, or muscle flaps may be employed to ensure a durable repair while minimizing the risk of recurrence and complications [11]. In our case, given that the patient was pediatric, we opted for a double-layer anatomical closure using absorbable sutures.

One of the key learning points from this case is the importance of recognizing limitations in clinical assessment in young children. Certain objective findings, such as pain localization and symptom progression, may be difficult to interpret in pediatric patients. This highlights the necessity of using a combination of clinical judgment, serial examinations, and imaging where available to aid diagnosis. Although obstruction may be absent, early imaging and inflammatory markers can provide crucial indications of strangulation, necessitating prompt surgical intervention. This underscores the importance of maintaining a high index of suspicion and ensuring timely management to prevent serious complications such as bowel necrosis and perforation [12]. Our case further reinforces that, in pediatric patients with recurrent hernia symptoms, early elective surgical repair should be strongly considered to avoid complications associated with delayed intervention.

This case is notable due to its clinical significance. While Richter’s hernia predominantly affects adults, particularly the elderly, our patient was only four years old. Furthermore, the umbilical location is an uncommon site for this hernia type, adding to its uniqueness. Despite initially mild symptoms, the patient developed bowel perforation, demonstrating how subtle presentations can mask severe pathology. Most pediatric cases of Richter’s hernia occur in the inguinal region, making this one of the few documented cases of congenital umbilical Richter’s hernia with bowel perforation [2,4,10,12].

## Conclusions

Richter’s hernia poses a diagnostic challenge, requiring a high index of suspicion, particularly in pediatric patients with non-reducible umbilical hernias. Early recognition and timely surgical intervention are crucial to preventing complications such as bowel necrosis, perforation, and fistula formation. Optimizing surgical management based on hernia size, anatomical location, and bowel viability is essential for improving patient outcomes and reducing recurrence. Ultimately, early diagnosis and prompt surgical treatment remain the cornerstone of effective management, preventing life-threatening complications.
